# Supplementary material for: A Machine Learning Ensemble Based on Radiomics to Predict BI-RADS Category and Reduce the Biopsy Rate of Ultrasound-Detected Suspicious Breast Masses
Source: Diagnostics (Basel). 2022 Jan 13;12(1):187. doi: 10.3390/diagnostics12010187 (PMC8774734; doi:10.3390/diagnostics12010187)
Supplement: Supplementary file 1 [file diagnostics-12-00187-s001.zip › diagnostics-1515725-supplementary.pdf]

Supplementary Material

Table S1. Ensemble of random forest classifiers. Classification performance and statistical significance with respect to chance/random classification (*p* value). Performances are reported for a majority vote of 50% and for the internal testing.

Metric	Internal testing
ROC-AUC (%) (95% confidence interval)	68** (67–69)
Sensitivity (%) (95% confidence interval)	64** (60–68)
Specificity (%) (95% confidence interval)	63** (62–64)
Positive predictive value (%) (95% confidence interval)	63** (62–64)
Negative predictive value (%) (95% confidence interval)	63** (62–65)

** denotes a statistical significance at 0.005 (adjusted with Bonferroni-Holm correction).

Table S2. Ensembles of support vector machine classifiers. Classification performances and statistical significance with respect to chance/random classification (*p* value). Performances are reported for a majority vote of 50% and for the internal testing.

Metric	Internal testing
ROC-AUC (%) (95% confidence interval)	75** (74–75)
Sensitivity (%) (95% confidence interval)	72** (71–73)
Specificity (%) (95% confidence interval)	70** (66–74)
Positive predictive value (%) (95% confidence interval)	71** (67–74)
Negative predictive value (%) (95% confidence interval)	71** (70–73)

** denotes a statistical significance at 0.005 (adjusted with Bonferroni-Holm correction).

Table S3. Ensembles of k nearest neighbors classifiers. Classification performances and statistical significance with respect to chance/random classification (*p* value). Performances are reported for a majority vote of 50% and for the internal testing.

Metric	Internal testing
ROC-AUC (%) (95% confidence interval)	74** (73–75)
Sensitivity (%) (95% confidence interval)	70** (67–72)
Specificity (%) (95% confidence interval)	67** (61–73)
Positive predictive value (%) (95% confidence interval)	68** (64–72)
Negative predictive value (%) (95% confidence interval)	69** (67–71)

** denotes a statistical significance at 0.005 (adjusted with Bonferroni-Holm correction).

Table S4. Complete list of 107 radiomic features with the values of the four representative lesions (two benign and two malignant) shown in Figures 3 and 4.

Feature family	Feature name	Unit	Figure 3a (benign)	Figure 3b (benign)	Figure 4a (malignant)	Figure 4b (malignant)
Morphology	Area	mm^2	37.489	143.634	214.474	60.387
Morphology	Perimeter	mm	24.165	50.155	68.826	35.145
Morphology	Perimeter to area ratio	mm	0.645	0.349	0.321	0.582
Morphology	Compactness	–	0.807	0.718	0.569	0.614
Morphology	Circularity	–	0.898	0.847	0.754	0.784
Morphology	Acircularity	–	0.113	0.181	0.326	0.276
Morphology	Center of mass shift	mm	3.495	4.458	3.997	5.861
Morphology	Maximum diameter	mm	8.934	16.264	21.153	13.951
Intensity-based statistics	Mean	–	11.369	62.602	29.547	67.277
Intensity-based statistics	Variance	–	140.133	426.847	500.680	364.277
Intensity-based statistics	Median	–	7	60	25	66
Intensity-based statistics	Minimum	–	0	10	0	15
Intensity-based statistics	10th percentile	–	2	38	5	44
Intensity-based statistics	90th percentile	–	27	89	60	90
Intensity-based statistics	Interquartile range	–	10	26	29	23
Intensity-based statistics	Mean absolute deviation	–	8.2323	15.9370	17.5030	14.4264
Intensity-based statistics	Robust mean absolute deviation	–	4.7193	10.9774	12.5114	9.5833

Intensity-based statistics	Median absolute deviation	–	7.4631	15.8155	17.1981	14.4122
Intensity-based statistics	Coefficient of variation	–	1.0412	0.3300	0.7573	0.2837
Intensity-based statistics	Quartile coefficient	–	0.5556	0.2131	0.5472	0.1729
Intensity-based statistics	Energy	–	869080	53714028	25352896	49809980
Intensity-based statistics	Root mean	–	16.413	65.923	37.063	69.932
Intensity histogram	Mean	–	9.675	17.789	11.821	23.116
Intensity histogram	Variance	–	89.795	45.926	73.689	68.073
Intensity histogram	Median	–	6	17	10	23
Intensity histogram	10th percentile	–	2	10	2	13
Intensity histogram	90th percentile	–	22	26	23	33
Intensity histogram	Maximum	–	64	64	64	64
Intensity histogram	Range	–	63	63	63	63
Intensity histogram	Mean absolute deviation	–	6.576	5.226	6.713	6.214
Intensity histogram	Robust mean absolute deviation	–	3.773	3.600	4.980	4.241
Intensity histogram	Median absolute deviation	–	5.983	5.183	6.583	6.201
Intensity histogram	Coefficient of variation	–	0.979	0.381	0.726	0.357
Intensity histogram	Entropy	–	4.494	4.738	4.806	5.004
Intensity histogram	Uniformity	–	0.065	0.045	0.044	0.039
Intensity histogram	Minimum histogram gradient	–	-157.5	-134.5	-332.0	-175.5

Grey-level co-occurrence matrix	Joint maximum	–	0.040	0.013	0.047	0.015
Grey-level co-occurrence matrix	Joint average	–	9.121	17.690	11.684	22.991
Grey-level co-occurrence matrix	Joint variance	–	75.650	44.367	71.977	66.033
Grey-level co-occurrence matrix	Joint entropy	–	7.830	8.218	7.879	8.619
Grey-level co-occurrence matrix	Difference average	–	2.843	2.217	1.858	2.473
Grey-level co-occurrence matrix	Difference variance	–	11.931	4.380	3.717	5.181
Grey-level co-occurrence matrix	Difference entropy	–	3.138	2.765	2.606	2.906
Grey-level co-occurrence matrix	Sum average	–	18.242	35.381	23.369	45.982
Grey-level co-occurrence matrix	Sum variance	–	282.587	168.172	280.738	252.834
Grey-level co-occurrence matrix	Sum entropy	–	5.420	5.686	5.807	5.978
Grey-level co-occurrence matrix	Angular second moment	–	0.0106	0.0051	0.0086	0.0041
Grey-level co-occurrence matrix	Contrast	–	20.012	9.294	7.169	11.297
Grey-level co-occurrence matrix	Dissimilarity	–	2.843	2.217	1.858	2.473
Grey-level co-occurrence matrix	Inverse difference	–	0.444	0.449	0.508	0.426
Grey-level co-occurrence matrix	Inverse difference normalised	–	0.960	0.967	0.973	0.964
Grey-level co-occurrence matrix	Inverse difference moment	–	0.373	0.377	0.446	0.349
Grey-level co-occurrence matrix	Inverse difference moment normalised	–	0.995	0.998	0.998	0.997
Grey-level co-occurrence matrix	Inverse variance	–	0.312	0.364	0.370	0.340
Grey-level co-occurrence matrix	Autocorrelation	–	148.839	352.667	204.915	588.976

Grey-level co-occurrence matrix	Cluster tendency	–	282.587	168.172	280.738	252.834
Grey-level co-occurrence matrix	First measure of information correlation	–	-0.223	-0.259	-0.354	-0.271
Grey-level run length matrix	Short run emphasis	–	0.874	0.879	0.841	0.894
Grey-level run length matrix	Long runs emphasis	–	1.966	1.735	2.379	1.668
Grey-level run length matrix	Low grey level run emphasis	–	0.0745	0.0060	0.0465	0.0038
Grey-level run length matrix	High grey level run emphasis	–	212.347	371.377	242.140	615.364
Grey-level run length matrix	Short run low grey level emphasis	–	0.0525	0.0052	0.0295	0.0033
Grey-level run length matrix	Short run high grey level emphasis	–	203.341	332.728	219.252	558.174
Grey-level run length matrix	Long run low grey level emphasis	–	0.312	0.011	0.307	0.007
Grey-level run length matrix	Long run high grey level emphasis	–	260.514	603.440	375.931	961.905
Grey-level run length matrix	Grey level non uniformity	–	608.175	1824.154	2270.620	1327.523
Grey-level run length matrix	Grey level non uniformity normalized	–	0.0581	0.0443	0.0402	0.0384
Grey-level run length matrix	Run length non uniformity	–	7548.2	30082.6	37346.9	26255.1
Grey-level run length matrix	Run length non uniformity normalized	–	0.722	0.731	0.662	0.759
Grey-level run length matrix	Run percentage	–	0.811	0.833	0.764	0.849
Grey-level run length matrix	Grey level variance	–	99.103	47.546	75.238	69.208
Grey-level run length matrix	Run length variance	–	0.445	0.294	0.668	0.280
Grey-level run length matrix	Run entropy	–	5.395	5.528	5.829	5.720
Grey-level size zone matrix	Small zone emphasis	–	0.718	0.636	0.625	0.672

Grey-level size zone matrix	Large zone emphasis	–	21.219	8.482	269.335	7.599
Grey-level size zone matrix	Low grey level zone emphasis	–	0.0381	0.0056	0.0196	0.0035
Grey-level size zone matrix	High grey level zone emphasis	–	299.7	401.7	313.5	657.7
Grey-level size zone matrix	Small zone low grey level emphasis	–	0.0192	0.0032	0.0086	0.0021
Grey-level size zone matrix	Small zone high grey level emphasis	–	260.663	273.737	223.044	465.417
Grey-level size zone matrix	Large zone high grey level emphasis	–	7.183	0.056	67.193	0.035
Grey-level size zone matrix	Grey level non uniformity glszm	–	69.409	252.892	259.508	194.439
Grey-level size zone matrix	Grey level non uniformity normalized glszm	–	0.0445	0.0425	0.0364	0.0369
Grey-level size zone matrix	Zone size non uniformity	–	743.6	2255.5	2600.2	2209.0
Grey-level size zone matrix	Zone size non uniformity normalized	–	0.477	0.379	0.365	0.420
Grey-level size zone matrix	Zone percentage glszm	–	0.484	0.482	0.386	0.517
Grey-level size zone matrix	Grey level variance glszm	–	122.985	52.887	79.570	73.058
Grey-level size zone matrix	Zone size variance	–	16.943	4.170	262.637	3.854
Grey-level size zone matrix	Zone size entropy	–	6.407	6.750	7.036	6.820
Neighbourhood grey tone difference matrix	Coarseness	–	0.0037	0.0015	0.0013	0.0022
Neighbourhood grey tone difference matrix	Contrast	–	0.0923	0.0390	0.0475	0.0410
Neighbourhood grey tone difference matrix	Busyness	–	0.521	0.545	1.052	0.243
Neighbourhood grey tone difference matrix	Strength	–	17.164	3.142	4.050	5.143
Neighbouring grey level dependence matrix	Low dependence emphasis	–	0.421	0.394	0.324	0.432

Neighbouring grey level dependence matrix	High dependence emphasis	–	8.809	6.908	11.245	6.241
Neighbouring grey level dependence matrix	Low grey level count emphasis	–	0.100	0.006	0.070	0.004
Neighbouring grey level dependence matrix	High grey level count emphasis	–	183.404	362.360	213.429	602.416
Neighbouring grey level dependence matrix	Low dependence low grey level emphasis	–	0.0163	0.0021	0.0075	0.0014
Neighbouring grey level dependence matrix	Low dependence high grey level emphasis	–	133.879	161.772	103.073	288.651
Neighbouring grey level dependence matrix	High dependence low grey level emphasis	–	2.165	0.044	2.032	0.026
Neighbouring grey level dependence matrix	Grey level non uniformity	–	211.159	555.511	812.533	397.161
Neighbouring grey level dependence matrix	Grey level non uniformity normalized	–	0.0655	0.0449	0.0440	0.0390
Neighbouring grey level dependence matrix	Dependence count non uniformity	–	737.2	3177.8	3651.0	2777.8
Neighbouring grey level dependence matrix	Dependence count non uniformity normalized	–	0.229	0.257	0.198	0.273
Neighbouring grey level dependence matrix	Grey level variance	–	89.795	45.926	73.689	68.073
Neighbouring grey level dependence matrix	Dependence count variance	–	2.486	1.446	2.925	1.354
Neighbouring grey level dependence matrix	Dependence count entropy	–	6.473	6.831	7.030	6.990
Neighbouring grey level dependence matrix	Dependence count energy	–	0.0158	0.0115	0.0098	0.0109

Table S5. BI-RADS classes assigned by the ensemble of support vector machines (AI model) and the certified breast radiologist

AI model	Certified breast radiologist
BI-RADS 3	BI-RADS 4
BI-RADS 4	BI-RADS 5
BI-RADS 4	BI-RADS 4
BI-RADS 4	BI-RADS 4
BI-RADS 4	BI-RADS 4
BI-RADS 4	BI-RADS 4
BI-RADS 4	BI-RADS 4
BI-RADS 4	BI-RADS 4
BI-RADS 4	BI-RADS 4
BI-RADS 3	BI-RADS 3
BI-RADS 3	BI-RADS 4
BI-RADS 4	BI-RADS 4
BI-RADS 4	BI-RADS 4
BI-RADS 3	BI-RADS 4
BI-RADS 3	BI-RADS 3
BI-RADS 3	BI-RADS 4
BI-RADS 4	BI-RADS 5
BI-RADS 4	BI-RADS 4
BI-RADS 3	BI-RADS 3
BI-RADS 4	BI-RADS 4
BI-RADS 4	BI-RADS 4
BI-RADS 4	BI-RADS 4
BI-RADS 4	BI-RADS 4
BI-RADS 4	BI-RADS 4
BI-RADS 4	BI-RADS 4
BI-RADS 4	BI-RADS 4
BI-RADS 4	BI-RADS 4
BI-RADS 4	BI-RADS 4
BI-RADS 4	BI-RADS 4
BI-RADS 4	BI-RADS 4

BI-RADS 4	BI-RADS 4
BI-RADS 4	BI-RADS 4
BI-RADS 4	BI-RADS 4
BI-RADS 4	BI-RADS 4
BI-RADS 4	BI-RADS 4
BI-RADS 3	BI-RADS 3
BI-RADS 4	BI-RADS 4
BI-RADS 4	BI-RADS 4
BI-RADS 4	BI-RADS 4
BI-RADS 4	BI-RADS 4
BI-RADS 4	BI-RADS 4
BI-RADS 4	BI-RADS 4
BI-RADS 3	BI-RADS 3
BI-RADS 4	BI-RADS 4
BI-RADS 4	BI-RADS 4
BI-RADS 4	BI-RADS 4
BI-RADS 4	BI-RADS 4
BI-RADS 4	BI-RADS 4
BI-RADS 4	BI-RADS 4
BI-RADS 4	BI-RADS 4
BI-RADS 4	BI-RADS 4
BI-RADS 4	BI-RADS 4
BI-RADS 4	BI-RADS 4
BI-RADS 4	BI-RADS 4
BI-RADS 4	BI-RADS 4
BI-RADS 4	BI-RADS 4
BI-RADS 4	BI-RADS 4
BI-RADS 4	BI-RADS 4
BI-RADS 4	BI-RADS 4
BI-RADS 4	BI-RADS 4
BI-RADS 4	BI-RADS 5
BI-RADS 4	BI-RADS 4
BI-RADS 4	BI-RADS 4
BI-RADS 4	BI-RADS 4

BI-RADS 4	BI-RADS 4
BI-RADS 4	BI-RADS 4
BI-RADS 4	BI-RADS 4
BI-RADS 4	BI-RADS 4
BI-RADS 3	BI-RADS 3
BI-RADS 4	BI-RADS 4
BI-RADS 4	BI-RADS 4
BI-RADS 4	BI-RADS 4
BI-RADS 4	BI-RADS 4
BI-RADS 4	BI-RADS 4
BI-RADS 4	BI-RADS 4
BI-RADS 4	BI-RADS 4
BI-RADS 4	BI-RADS 4
BI-RADS 4	BI-RADS 4
BI-RADS 4	BI-RADS 4
BI-RADS 4	BI-RADS 4
BI-RADS 4	BI-RADS 4
BI-RADS 4	BI-RADS 4
BI-RADS 4	BI-RADS 4
BI-RADS 4	BI-RADS 4
BI-RADS 4	BI-RADS 4
BI-RADS 4	BI-RADS 4
BI-RADS 4	BI-RADS 4
BI-RADS 4	BI-RADS 4
BI-RADS 4	BI-RADS 4
BI-RADS 4	BI-RADS 4
BI-RADS 4	BI-RADS 4
BI-RADS 4	BI-RADS 4
BI-RADS 4	BI-RADS 4
BI-RADS 4	BI-RADS 4
BI-RADS 4	BI-RADS 4
BI-RADS 4	BI-RADS 4
BI-RADS 4	BI-RADS 4
BI-RADS 4	BI-RADS 4

BI-RADS 4	BI-RADS 4
BI-RADS 3	BI-RADS 4
BI-RADS 3	BI-RADS 4
BI-RADS 4	BI-RADS 4
BI-RADS 4	BI-RADS 4
BI-RADS 4	BI-RADS 4
BI-RADS 4	BI-RADS 4
BI-RADS 4	BI-RADS 4
BI-RADS 4	BI-RADS 4
BI-RADS 3	BI-RADS 3
BI-RADS 3	BI-RADS 3
BI-RADS 4	BI-RADS 4
BI-RADS 4	BI-RADS 4
BI-RADS 4	BI-RADS 4
BI-RADS 4	BI-RADS 4
BI-RADS 4	BI-RADS 4
BI-RADS 4	BI-RADS 4
BI-RADS 4	BI-RADS 4
BI-RADS 4	BI-RADS 4
BI-RADS 4	BI-RADS 4
BI-RADS 4	BI-RADS 4
BI-RADS 4	BI-RADS 4
BI-RADS 4	BI-RADS 4
BI-RADS 4	BI-RADS 4
BI-RADS 4	BI-RADS 4

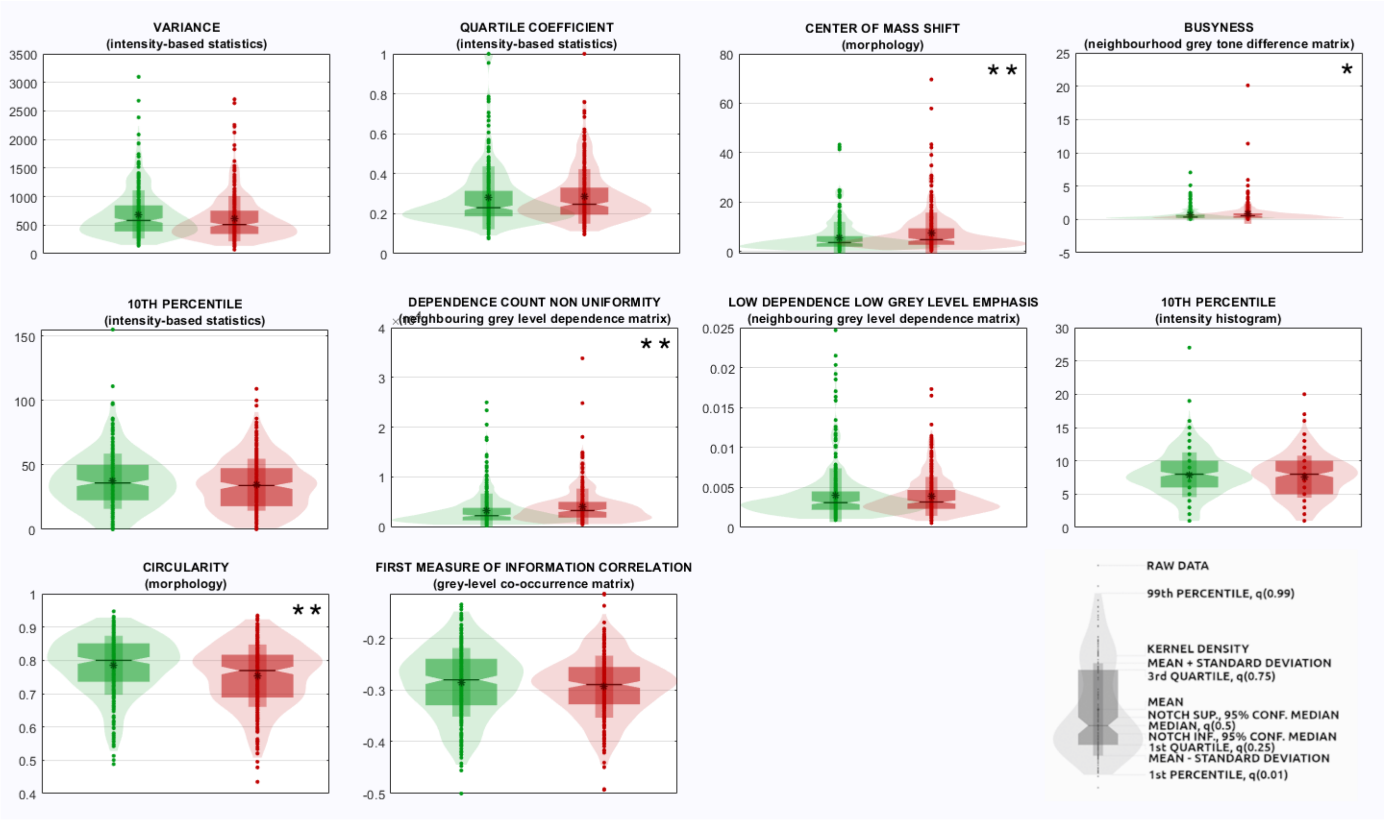

Figure S1. Violin and box plots of the most relevant features ranked from 16 to 25
Green: benign class. Red: malignant class.
* denotes statistical significance at 0.05 (adjusted with Bonferroni-Holm correction). ** denotes a statistical significance at 0.005 (adjusted with Bonferroni-Holm correction).
